# Flavonoids in the Spotlight: Bridging the Gap between Physicochemical Properties and Formulation Strategies

**DOI:** 10.3390/ph16101407

**Published:** 2023-10-03

**Authors:** Marta Berga, Konstantins Logviss, Liga Lauberte, Artūrs Paulausks, Valentyn Mohylyuk

**Affiliations:** Laboratory of Finished Dosage Forms, Faculty of Pharmacy, Riga Stradiņš University, LV-1007 Riga, Latvia

**Keywords:** flavonoids, physiochemical properties, solubility, p*K*_a_, log *P*, permeability, solubility parameters, brick dust, grease balls, developability classification system

## Abstract

Flavonoids are hydroxylated polyphenols that are widely distributed in plants with diverse health benefits. Despite their popularity, the bioavailability of flavonoids is often overlooked, impacting their efficacy and the comparison of products. The study discusses the bioavailability-related physicochemical properties of flavonoids, with a focus on the poorly soluble compounds commonly found in dietary supplements and herbal products. This review sums up the values of p*K*_a_, log *P*, solubility, permeability, and melting temperature of flavonoids. Experimental and calculated data were compiled for various flavonoid subclasses, revealing variations in their physicochemical properties. The investigation highlights the challenges posed by poorly soluble flavonoids and underscores the need for enabling formulation approaches to enhance their bioavailability and therapeutic potential. Compared to aglycones, flavonoid glycosides (with sugar moieties) tend to be more hydrophilic. Most of the reviewed aglycones and glycosides exhibit relatively low log *P* and high melting points, making them “brick dust” candidates. To improve solubility and absorption, strategies like size reduction, the potential use of solid dispersions and carriers, as well as lipid-based formulations have been discussed.

## 1. Introduction

Scientific evidence is necessary to support the importance of the bioavailability of nutraceutical/pharmaceutical substances, especially those that are poorly soluble, such as flavonoids. Many flavonoids currently used as dietary supplements or medical devices are poorly soluble and demonstrate poor bioavailability [1]. In 2021, according to the Nutrition Business Journal, U.S. herbal dietary supplement retail sales were worth about USD 12.350 billion. That is a 10% increase in total sales compared to the previous year and is the second highest for these products after 2020’s record increase of 17% from 2019 [2]. Unfortunately, the bioavailability of herbal supplements is often overlooked, which complicates the assessment of their dose-related effects and the comparison of marketed products. At the same time, the pharmaceutical industry and regulatory authorities (European Medicines Agency and U.S. Food and Drug Administration) have already developed approaches to the assessment and classification of poorly soluble drugs (e.g., biopharmaceutical classification system), which could be used and applied to flavonoids with non-medical claims [3,4].

The aim of this investigation is to use a representative list of flavonoids; overview their bioavailability-related physiochemical properties p*K*_a_, log *P*, solubility, solubility parameters, melting temperature, and permeability; and discuss the formulation-related attributes of flavonoids in the context of their bioavailability.

## 2. Discussion

### 2.1. Flavonoids—Classification, Health Effects, Applications

Hydroxylated polyphenols, also called flavonoids, are a group of secondary plant metabolites that are abundantly present throughout the whole plant kingdom, including cereals, fruits, vegetables, herbs, seeds, and flowers of numerous plants, which are important dietary sources. Flavonoids are widely distributed in all plant parts, including the roots, leaves, and shoots. In the literature, 8000–10,000 compounds of polyphenols have been identified as flavonoids [5,6]. Numerous studies have revealed that flavonoids have several biological effects and properties [7,8,9,10,11]. The properties of flavonoids include antiviral activities, particularly against various types of human herpesviruses such as tumor herpesviruses. Flavonoids have antiallergenic and anticancer effects, as well as the potential to aid in the development of personalized, preventive, and predictive medicine. Thanks to these health benefits, more people are taking flavonoids not only with food but also as dietary supplements—single purified compounds and/or complex mixtures of compounds [6,12,13,14]. It has been reported that the mean intake of flavonoids from different foods in Europe was 428 ± 49 mg per day. Different food choices by country and, consequently, different preferences for the primary dietary sources may influence flavonoid consumption. Plants that contain flavonoids have also been used medicinally for a long time [15].

**Figure 1:**
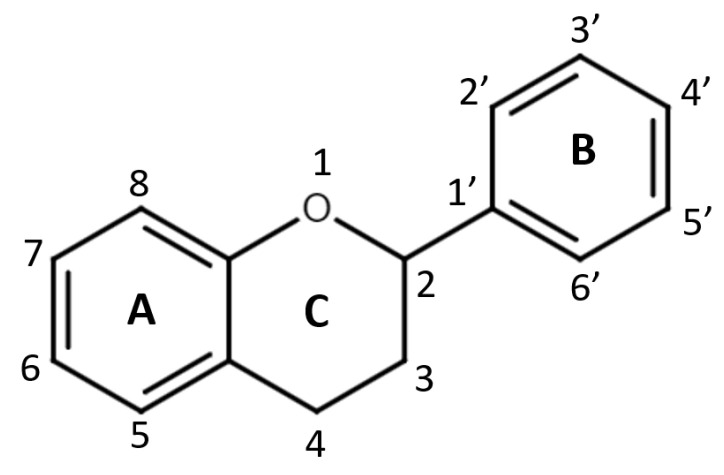
The general skeleton structure of flavonoids.

Regarding the chemistry of these hydroxylated polyphenols, the word “flavonoid” can be applied to compounds in three cases: (1) compounds with a C15 skeleton (also known as C_6_-C_3_-C_6_ backbone structure) that are derivatives of phenyl-substituted propylbenzene; (2) phenyl-substituted propylbenzene derivatives with a C16 backbone (rotenoids, mainly used as insecticides); (3) compounds with a structure that is based on phenyl-substituted propylbenzene derivatives condensed with C_6_-C_3_ precursors, also known as flavonolignans [16]. Flavonoids consisting of 15 carbon atoms are structured into two 6-carbon rings (referred to as the A- and B-rings) joined by a heterocyclic benzopyran C-ring that contains oxygen (Figure 1). Based on the position of the B-ring, as well as saturation and oxidation of the C-ring, flavonoids are classified into eight subclasses: flavones, isoflavones, flavonols, chalcones, anthocyanidins, flavanones, flavanols (flavan-3-ols), and flavanonols. Furthermore, flavonoids in natural products exist not only as aglycones (the parent cyclic structures, free form) but also as β-glycosides (*O*- and *C*-glycosylated derivatives) [13,16]. Many studies conducted previously have focused mostly on compounds with a C15 skeleton and have rarely included flavonolignans or did not include compounds from all eight subclasses.

Flavones, found in the leaves, flowers, and fruits of plants such as mint and chamomile, are a subgroup of compounds with a ketone in position 4 of the C ring and a double bond between positions 2 and 3. The main and more commonly described compounds of this subgroup are apigenin and luteolin (their glycosides, apigetrin and cynaroside) [17]. Additionally, for many years, there has been interest in baicalin and its aglycone, baicalein, a major flavonoid found in the rhizomes of the traditional Chinese medicinal herb *Scutellaria baicalensis* L. [18,19,20].

Flavonols are ketone-containing flavonoids found in an assortment of vegetables (e.g., onions) and fruits (e.g., apples and berries), as well as in beverages (tea and wine). The most studied compounds in this group are quercetin, myricetin, fisetin, and kaempferol. Quercetin should especially be emphasized in this group, as it is at the top of the list of best-selling dietary supplements [21,22]. Consumption of flavonols has been linked to a number of health advantages, including antioxidant activity and a decreased risk of vascular disease. Additionally, a quercetin glycoside called quercetin 3-O-rutinoside (rutin) is a well-liked flavonoid supplement that is frequently combined with quercetin dosages [23]. Thus, rutin trihydrate (also known as rutoside trihydrate), in addition to trypsin and bromelain, is a medication used to relieve musculoskeletal pain in conditions such as osteoarthritis and rheumatoid arthritis [24,25].

Isoflavones are isomers of flavones that are dominantly found in leguminous plants and are mostly extracted from soybeans. Commonly found isoflavones are the aglycones daidzein and genistein and the glycosides daidzin and genistin. Isoflavones are referred to as phytoestrogens because of their structural and biological similarities to the female hormone estrogen. It has been discovered that phytoestrogens may be used as an alternative therapy for hormone-dependent diseases such as cancer, menopausal syndrome, cardiovascular disorders, and osteoporosis. By boosting bone density, isoflavones also contribute to bone health and bone strength [26].

Furthermore, the flavanones have the C-ring saturated and can also be called dihydroflavones. Unlike flavones, these compounds have a saturated double bond between positions 2 and 3. Flavanones are mostly associated with oranges, lemons, and grapefruits, as they are present in all citrus fruits and are responsible for the bitter taste in juice and peel. The most known compounds of this subclass are hesperetin and naringerin, but even better known is a combination of two compounds: hesperidin (hesperetin 7-O-β-rutinoside) and diosmin (diosmetin 7-O-rutinoside). Although diosmin and its aglycone diosmetin belong to flavones, this combination is widely presented in supplements and is used in authorized medicine (Daflon^®^). Citrus flavonoids have intriguing pharmacological properties such as antioxidant and anti-inflammatory properties; they can also fulfill the role of blood lipid-lowering and cholesterol-lowering agents [17,27].

Flavanols, also called as “flavan-3-ols” or “catechins”, have a hydroxyl group at position 3 but lack both the ketone group and the saturated bond between positions 2 and 3. Catechins are widely found in fruits and berries as well as herbal plants [13,28]. Flavanonols, also called “dihydroflavonols,” are limitedly distributed in citrus fruits and *Glycosmis* L. species [29]. Taxifolin, engeletin, and puyanol are the representatives of this class.

Chalcones are characterized by the absence of the so-called “C-ring” of the basic flavonoid structure. This group can also be referred to as open-chain flavonoids. Representatives of this group are phloretin, phlorizin, arbutin, and more. Chalcones occur in vegetables, fruits and berries such as tomatoes, pears, and strawberries [17]. Anthocyanidins are groups of compounds characterized by the lack of the ketone group and by having two double bonds between positions 1 and 2, as well as between 3 and 4 (C-ring). Representatives such as delphinidin, malvidin, and cyanidin are found in flowers and berries [13].

**Figure 2:**
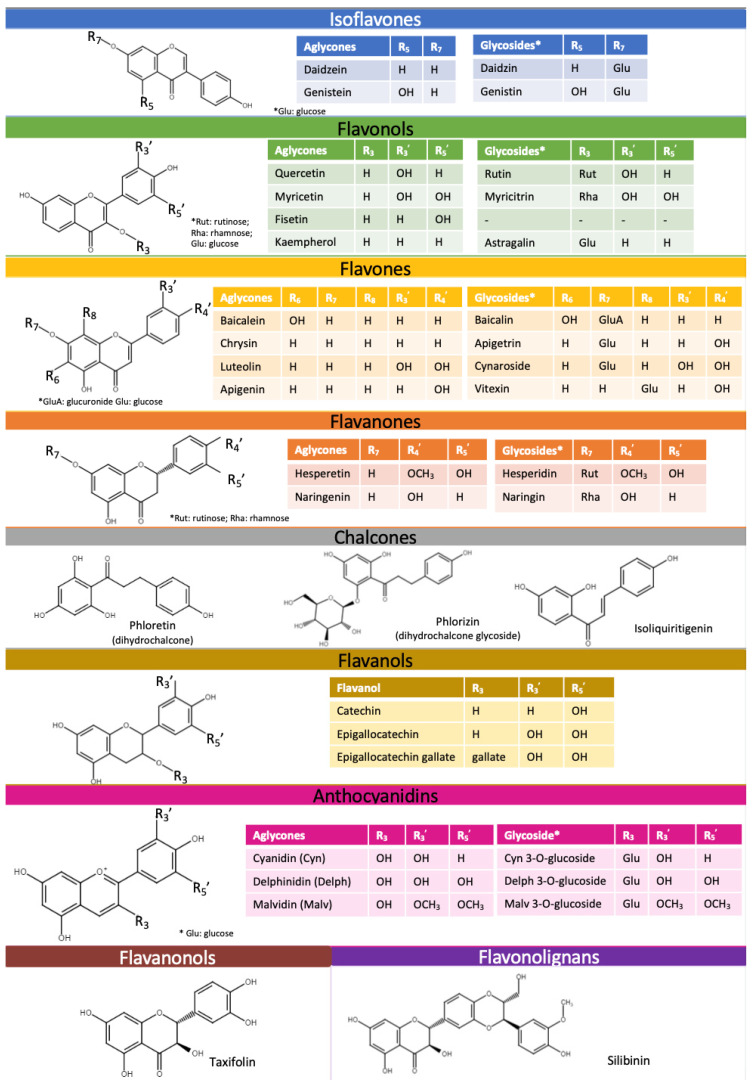
Structures of reviewed flavonoids (aglycones and glycosides) organized by their subclass. This color classification is utilized in all graphs throughout this review.

A popular compound among flavonolignans is silibinin. Silibinin is found in sylimarin, which is a common extract from the seeds of milk thistle (*Silybum marianum *L.). Currently, the main uses of milk thistle are for hepatoprotection, the prevention and therapy of liver disease and hepatic injury [30]. Figure 2 describes the structure and classification of the monomeric flavonoids and glycosides (reviewed in the paper), utilizing the framework for flavonoid structural organization [31].

### 2.2. Acid-Base Dissociation Constant (pK_a_)

The p*K*_a_ of a compound significantly impacts biopharmaceutical attributes such as solubility, lipophilicity, permeability, and protein binding. Understanding the dissociation constant of weak acid and base drugs is crucial for determining their ionic state across various pH levels [32]. Despite recent attention on flavonoids, there is a lack of comprehensive knowledge about their experimental p*K*_a_ values and other physicochemical properties. The likely reason for this is that they are mostly extracted and used as a sum of compounds but not as highly purified compounds. The molecular complexity of flavonoids also contributes to this issue [33]. Computer-aided calculations in cheminformatics and computational pharmacology are widely used to determine new drug-like molecules, saving time, human effort, and resources, especially when experimental data are unavailable [34]. However, these predicted values rarely match the experimentally determined data due to a variety of factors. Additionally, it is impossible to distinguish the range of acidity of certain -OH groups in polyhydroxylated phenols using spectrophotometric or potentiometric titrations. To the best of our knowledge, only a few studies have actually addressed the location of the main dissociation [35]. Since experimental values are missing for some representatives of aglycones and several representatives of glycosides, this is an area for potential research.

**Figure 3:**
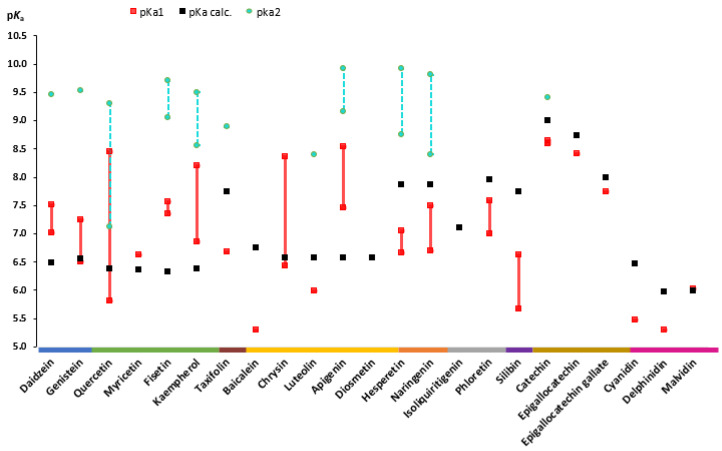
Experimental and theoretically calculated p*K*_a_ values of aglycones (based on the data from Table A1 [30,33,35,38,39,40,41,42,43,44,45,46,47,48,49,50,51,52,53,54,55,56,57,58]).

**Figure 4:**
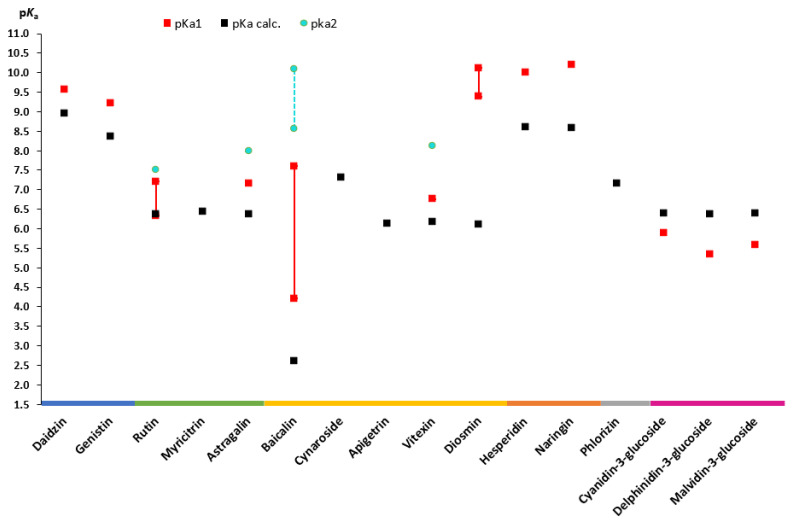
Experimental and theoretically calculated p*K*_a_ values of glycosides (based on the data from Table A1 [18,20,40,42,50,53,54,58,59,60,61,62,63,64]).

During the review, experimental data from different sources as well as calculated values of aglycones (Figure 3) and flavonoid glycosides (Figure 4) were collected. An analysis of the literature on dissociation constants reveals significant variation among published values for most flavonoids. Most often, the experimental p*K*_a_ values of flavonoids found in the literature are scattered and appear as secondary aspects in broader studies. One of the exceptions is a recently published paper by Fuguet et al. [33]. Due to this reason, multiple experimental values were plotted as ranges of values. Only p*K*_a_ values that are partially or fully ionized within a physiological pH (approximately up to 7.5) were included in the plots [36,37]. For many of the chosen flavonoids, dissociation constants exceeded the pH range mentioned above; thereby, mainly the p*K*_a1_ was included, and for individual compounds, the p*K*_a2_ was also included. No experimental values for aglycones, such as diosmetin and isoliquiritigenin, and glycosides, such as myricitrin, cynaroside, phlorizin, and apigetrin, were found in the literature.

Isoflavonones, flavonols, and flavones are all around the same p*K*_a_ value (6.5) according to calculated p*K*_a_ values. However, experimentally determined p*K*_a1_ values for the same compounds range from 5.30 (baicalein) to 8.54 (apigenin). The largest range of p*K*_a1_ values 5.81–8.45 was observed for quercetin, a flavonol. This is due to the fact that quercetin and its derivatives are among the most common compounds in a large number of plants. Quercetin comprises approximately 60–75% of the total intake of dietary flavonoids, and many biological studies have been carried out in the last few years including physical parameters [65,66]. Another reason for such a wide range can be the variety of determination methods (spectrophotometry, potentiometry, and capillary zone electrophoresis) and method/working conditions (solvents, concentrations, and compound solubility) used [33,67,68].

When comparing flavanols to other flavonoid subclasses, the dissociation constants are higher. The calculated p*K*_a_ of catechin, epigallocatechin (EGC), and epigallocatechin gallate (EGCG) is 9.00, 8.73, and 7.99, respectively. The minimal experimental p*K*_a1_ values of the same compounds are 8.60, 8.41, and 7.68, respectively. A drop in p*K*_a_ values is caused by the additional OH group that creates the pyrogallol moiety in the EGC structure and the additional galloyl moiety of the catechin molecule in EGCG [43,57].

The plot of aglycone p*K*_a_’s presented also remarks that the experimental values of anthocyanidins are in the range from 5.3 (delphinidin) to 6.0 (malvidin). In addition, the values of the glucosides of the same anthocyanidin aglycones are in a similar range (5.30–6.02).

Regarding the glycosides, it is worth mentioning baicalin, as it is one of the well-described glycosides in the literature with experimental constant values. Thus, the p*K*_a_ values for this compound are also in the largest range with p*K*_a1_ in the range from 4.21 to 7.6 and p*K*_a2_ in the range from 8.56 to 10.1 [18,20]. The calculated dissociation constant for this compound is 2.62, which means that baicalin can be ionized since entering the stomach environment.

Apart from anthocyanidins, it is clear that the experimentally determined p*K*_a1_ values are higher than the calculated values for most glycosides (Figure 4). Only a few glycosides, notably rutin, baicalin, and diosmin, have ranges, unlike aglycones, where most glycosides’ p*K*_a_ values are individual values that can be seen on the graph.

Based on the findings on dissociation constant data, it can be concluded that flavonoids are weak acidic molecules that are nonionized in the stomach under fasted conditions (pH 1–2). However, a small fraction of a few compounds including baicalein, cyanidin, delphinidin and baicalin can be ionized in postprandial conditions (pH up to 4). Moreover, most of the flavonoids are partially or to a certain extent ionized in the intestines, where the pH is 6.8–7.4.

### 2.3. Solubility and Permeability

Since 1995, when Amidon et al. introduced a relationship between human jejunal permeability rate measures (*P*_eff_) and the fraction of dosage absorbed, the biopharmaceutical classification system (BCS) has become a useful tool in drug development. According to their aqueous solubility and intestinal permeability, compounds are categorized into four classes [69,70]. Whereas compounds of high solubility, such as Class III and Class I, with low and high permeability, are relatively unproblematic, poorly soluble compounds of Class II and IV with high and low permeability are challenging cases to be delivered by oral route. In 2010, Butler and Dressman [71] developed a revised BCS and termed it the developability classification system (DCS), where Class II was further divided into dissolution rate-limited (IIa) and solubility-limited (IIb). For more realistic volumes of fluid available in the gastrointestinal tract and the compensatory nature of permeability on low solubility, the volume of 500 mL (DCS vs. 250 in BCS) was considered for the dose/solubility ratio [71]. To the best of our knowledge, only a few studies have reported flavonoids in the context of BCS, but as a sum of components and not as individual flavonoids [72,73].

**Figure 5:**
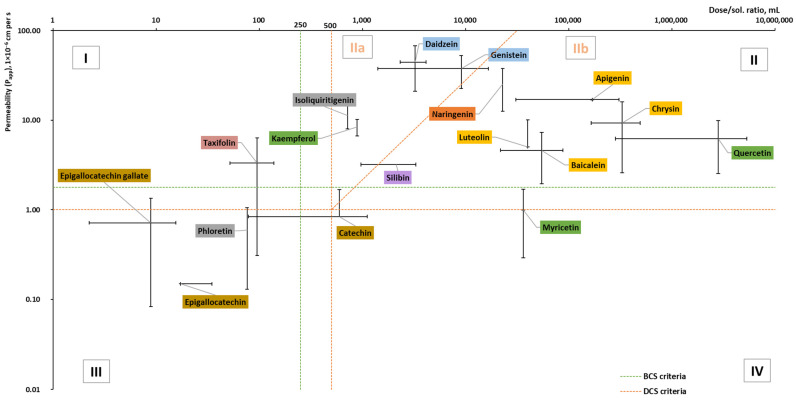
Dose/solubility ratio and permeability (*P*_app_) values of aglycones (based on the data from Table A2 [30,38,42,44,54,72,77,78,79,80,81,82,83,84,85,86,87,88,89,90,91,92,93,94,95,96,97,98,99,100,101,102,103,104,105,106,107,108,109,110,111,112,113,114,115,116,117,118,119,120,121,122,123,124]). BCS and DCS criteria based on [69,71,125].

**Figure 6:**
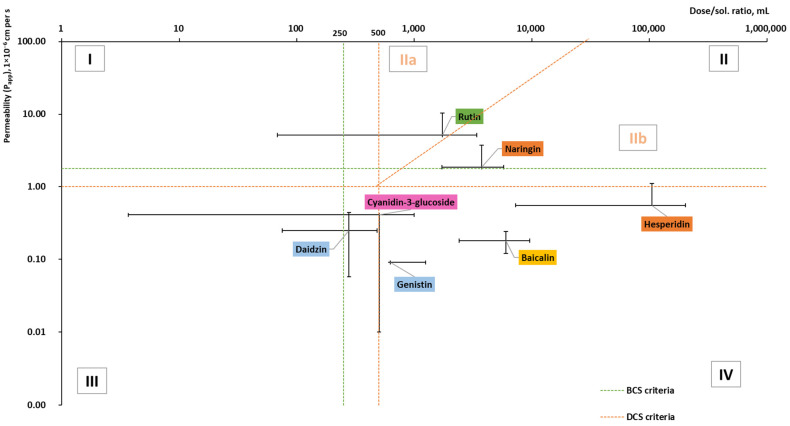
Dose/solubility ratio and permeability (*P*_app_) values of glycosides (based on the data from Table A2 [42,54,62,77,78,79,81,82,86,103,126,127,128,129,130,131,132,133,134,135,136,137,138,139,140,141,142,143,144,145,146,147]).

There have been multiple instances of published measurements for the *P*_eff_ of the human intestinal barrier, focusing on various pharmaceuticals. These measurements were attained by employing a single-pass perfusion method in the proximal jejunum. Due to the complex nature and significant expenses associated with in situ experiments, ongoing endeavors are directed toward simulating the in vivo system through in vitro apparent permeability (*P*_app_) measurements. For instance, Caco-2 cultured cell lines (monolayers) are utilized to model drug absorption in humans [74,75,76]. In this review, we used *P*_app_ (apical-basolateral) data obtained experimentally (Figure 5 and Figure 6), as they were available for the majority of flavonoids of interest, in contrast to *P*_eff_ data, which were frequently lacking. It should be noted that *P*_app_ data from Caco-2 cell monolayer models have been used in numerous studies [74,75,76] to predict oral drug absorption in humans (*P*_eff_). Although in vitro *P*_app_ values show a good correlation with in situ *P*_eff_ values, they cannot be directly equated due to the list of factors.

Based on the collected data (Figure 5), the reviewed aglycones belong to Classes II and III with the exception of taxifolin and myricetin. Taxifolin is in Class I but is approaching Classes II and III, whereas myricetin belongs to Class IV but is on the borderline of Class II. In addition, all reviewed flavanols belong to Class III, whereas all flavones belong to Class II. For certain aglycones such as hesperetin and diosmetin, the permeability has been determined; however, due to missing doses (there is no information on dietary supplements (DS), as in DS and medicines, the glycosides of these compounds are used), they were not included in the graph.

Glycosides are mostly in classes II and IV, but only half of all reviewed glycosides were included in the graph (Figure 6). Myricitrin, astragalin, cynaroside, apigetrin, delphinidin 3-glucoside, and malvidin 3-glucoside lacked permeability and dose data. No permeability studies were performed for vitexin or phlorizin.

### 2.4. Solubility Parameters

**Figure 7:**
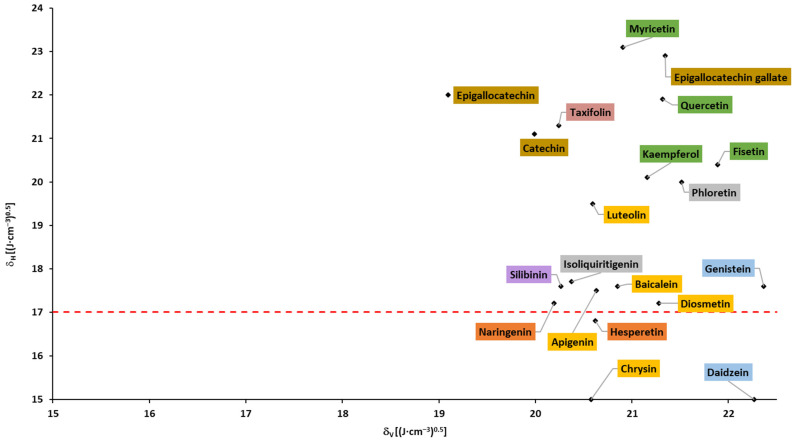
Bagley plot for aglycones (based on the data from Table A3).

**Figure 8:**
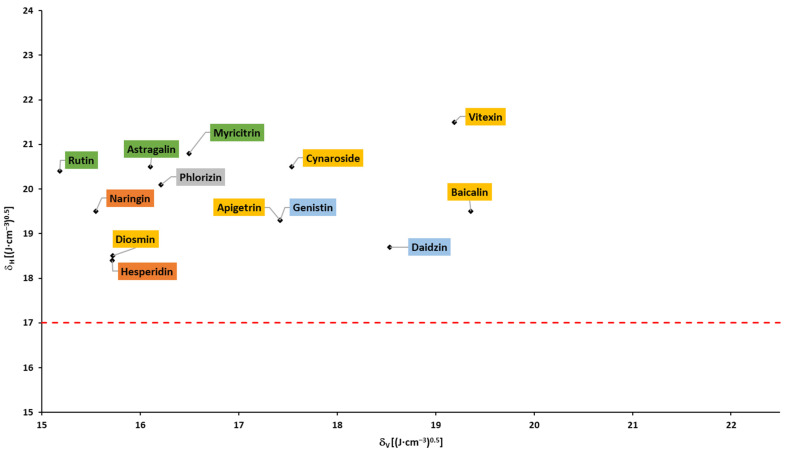
Bagley plot for glycosides (based on the data from Table A3).

Based on the Hansen solubility parameters, calculated with the Fedors and Van Krevelen/Hoftyzer group contribution method, Breitkreutz [148] proposed a Bagley plot (δH vs. δV; please see the Section 3). In accordance with the findings, drugs in a circular region with a center of 1.3 and 20.3, and a radius of 3$\sqrt{\frac{J}{{\mathrm{cm}}^{3}}}$, had absorption times >10 h and were absorbed within the whole gastrointestinal tract. Outside this circular region, at higher δH and/or δV but below δH of 17, the absorption times were 4–9 h. Drugs above δH of 17 were absorbed in the upper part of the gastrointestinal tract with an absorption time ≤3 h. In accordance with the abovementioned findings, none of the flavonoids fell into the circular region, and almost all flavonoids fell into region with δH > 17 (above the dotted line; Figure 7 and Figure 8), corresponding to the absorption in the upper parts of the small intestine and shorter absorption times. It can be observed that aglycones and glycosides are arranged in two areas that do not overlap.

Based on the Breitkreutz findings and presented results, an absorption window in the upper part of the small intestine and a limited (up to 3 h) absorption time of all flavonoids can be expected.

### 2.5. The Octanol/Water Partition Coefficient (K_O/W_) and Melting Point (T_m_)

The *K_O/W_* and *T_m_* are used for solubility calculation in accordance with the General Solubility Equation proposed by Jain and Yalkowsky [149]:

$$\log S_{W}^{solid}=0.5-0.01\;\left(T_{m}-25\right)-\log K_{O/W}$$

where $S_{W}^{solid}$ is solubility of the compound in water. Upon limited information, this equation can be used to predict the molar solubility and categorize compounds with poor, intermediate, or good solubility [150].

*K_O/W_*, or its logarithmic expression (log *P*), is one of the key parameters in drug discovery, design, and development [150]. Whereas water media can represent aqueous physiological media, the trivially employed amphiphilic n-octanol is considered to mimic the characteristics of the phospholipid membrane [151].

To differentiate formulation strategies based on the properties of the poorly soluble compounds such as log *P* and *T_m_*, the “grease ball/brick dust” classification was proposed and is currently widely used. The log *P* and *T_m_* are used to characterize compounds’ lipophilicity and crystal lattice strength, respectively. A relatively low lipophilicity (log *P* < 3) and a *T_m_* higher than 100 and 200 °C are used to classify the compound as “brick dust”. Another group, so-called “grease balls”, is characterized by relatively high lipophilicity (log *P* > 3) and substantially lower melting temperatures [152,153,154,155]. For example, while “grease balls” are more likely to be included in the lipid-based formulation, the “brick dust” candidates can be considered for polymer-based amorphous solid dispersions [156].

The same as with p*K_a_* values, the experimentally determined distribution coefficients vary between publications, and log *P* values for flavonoids are quite scattered as it is one of the parameters that are typically determined as a secondary or additional parameter [157,158]. In addition, most publications provide log *P* values for flavonoids, which have been calculated using one or various of the calculation tools, for example, SwissADME and ChemBioDraw Ultra 11.0 [34,159].

**Figure 9:**
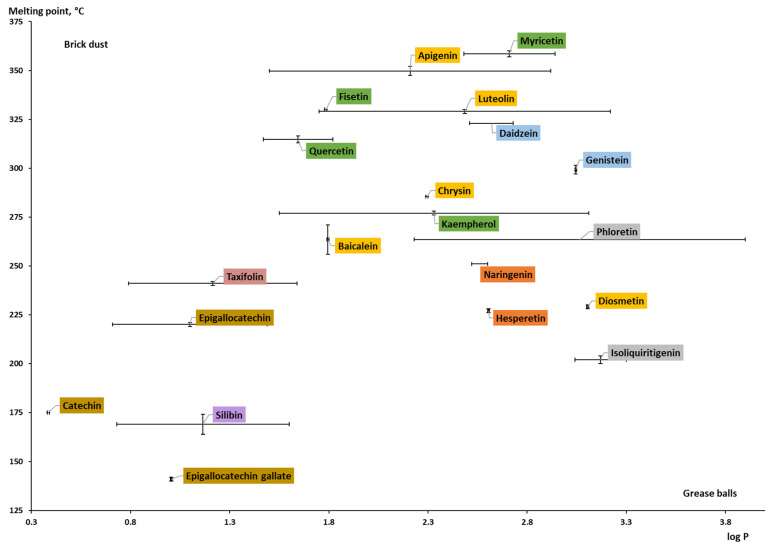
Experimental and theoretically calculated log *P* values and melting point of aglycones (based on the data from Table A1 [30,38,42,43,44,48,157,161,162,163,164,165,166,167,168,169,170,171,172,173]).

Plotting the collected log *P* against the melting point data (Table A1; Figure 9), it can be concluded that most studied flavonoid aglycones belong to the “brick dust” category, and only a few compounds (chalcones, phloretin, and isoliquiritigenin; flavone diosmetin) are moving toward the “grease balls” category. The most hydrophilic compounds (with relatively low log *P*) of the aglycones are the representatives of flavanols: catechin, epigallocatechin, epigallocatechin gallate, and flavonolignan silibinin. The representatives of each subclass are spotted in relative proximity to each other and can be circled as areas. For the compounds isoliquiritigenin, phloretin, and epigallocatechin, the calculated log *P* values were used since the experimental values were not available. Phloretin has the widest range regarding the partition coefficient value. It can be characterized by different calculation parameters. Additionally, the way the experiment was set up, the substance used, etc., can help explain why the ranges of values for other compounds, such as kaempferol, apigenin, and luteolin, whose values are determined experimentally, are so large. The log *P* values of the reviewed aglycones are within the range of 0.4–3.2. Regarding the *T_m_* of the aglycones, the values differ from 141 °C to 358 °C, and it can be said that each subclass of flavonoids is characterized by a certain melting temperature range. Flavanols have a lower range of *T_m_* (141–220 °C), while flavanones have a *T_m_* ranging from 227 °C to 251 °C. The melting point for isoflavones, flavonols, and flavones is over 250 °C.

Because the *T_m_* of anthocyanidins (both aglycones and glycosides) could not be found or were questionable, they were not included in the graphs. A few sources reported values that were used as beginning points or comparisons rather than as precise values. For instance, it was said that cyanidin had a *T_m_* of >300 °C (see Table A1). Thus, this subgroup was not graphically described.

**Figure 10:**
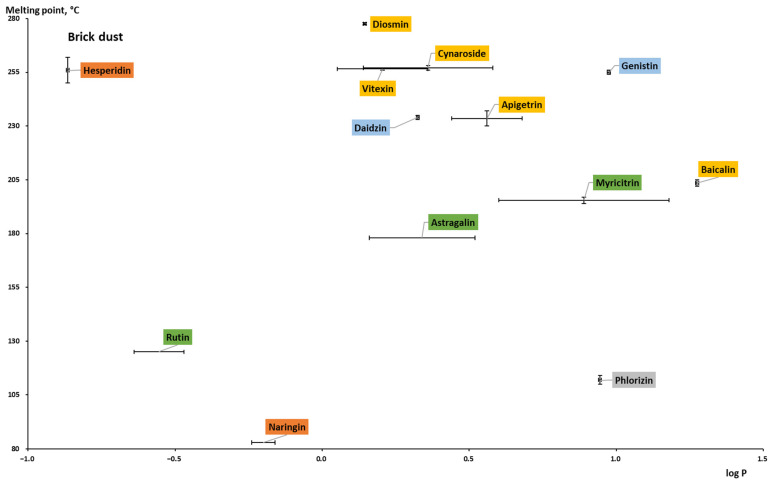
Experimental and theoretically calculated log *P* values and melting point of glycosides (based on the data from Table A1 [20,42,157,161,162,164,170,171,172,174,175,176,177,178,179,180,181]).

When the log *P* values of aglycones and glycosides are compared (Figure 9 and Figure 10), glycosides have a lower partition coefficient (maximum of 1.3 for baicalin), which means that aglycones are more lipophilic. The reason behind this is that log *P* for a sugar moiety is much lower than for the -OH group. For example, the log *P* for a glucose substituent is reported as roughly −2.4 [160], while for the hydroxyl group, it was calculated as −0.27 using the Molinspiration online interactive model. It is worth noting that the presence of a sugar moiety has a significant impact on the overall structure [157]. The log *P* values for naringin, hesperidin, and rutin are all negative. Furthermore, it is evident that in contrast to aglycones of flavanones, glycosides of the same subclass are distributed around the plot. Experimental data for glycosides have not been found; therefore, only calculated log *P* values were given for several conjugated flavonoids, including myricitrin, astragalin, cynaroside, apigetrin, vitexin, and naringin.

To be permeated and consequently absorbed, the poorly soluble flavonoids should be dissolved and made available in the small intestine where the permeation takes place. Oral formulation improvement strategies for flavonoids can be handled in numerous ways, such as structural transformation and chemical modification (glycosylation, metabolic conjugation, prenylation, “prodrugs”), absorption enhancers, as well as formulation and technological processing (e.g., cocrystals, nanotechnology) [1,182,183]. From the pharmaceutical technology side, the BCS/DCS and “brick dust/grease balls” classifications can help to choose the formulation approach. Most flavonoids were classified as “brick dust” (relatively high *T_m_* and low log *P*), while a few were classified as “grease balls.” Class I and III “brick dust” compounds are relatively unproblematic and can be formulated as immediate- or fast-release dosage forms. For Class IIa “brick dust” flavonoids whose dissolution rates are limited, such as daidzein, genistein, kaempferol, and rutin, the simplest way to increase the bioavailability is to increase the dissolution rate via particle size reduction. Whereas for solubility-limited Class IIb “brick dust” flavonoids, such as naringenin, apigenin, quercetin, and glycosides, the formulation should consider the increased apparent solubility and can include the solid-state modification (including solid dispersions and amorphous solid dispersions) [184]. These solid dispersions can be prepared by hot-melt extrusion, spray-drying, or electrospinning, as well as by the loading of flavonoids onto mesoporous carriers as can be exemplified by silybin (or Silymarin) [185]. For poorly soluble “grease balls” (relatively low *T_m_* and high log *P*), bioavailability can be enabled using different techniques. The simplest bioavailability-improving approach can be considered the co-consumption of fat-containing foods. The other approach is lipid-based formulations, including self-emulsifying (or self-micro-emulsifying) drug delivery systems (SEDDS/SMEDDS), micro-/nano-emulsions, and liposomes. Regarding Class IV compounds flavonoids (such as myricetin and the glycosides genistein, baicalin, and hesperidin), the same formulation approaches as for Class IIb “brick dust” and “grease balls” may be considered; however, cellular efflux issues should also be brought to attention [186]. For example, while the bioavailability of “brick dust” flavonoid glycoside hesperidin was improved by micronization [187], at the same time, in accordance with DCS, it is related to Class IV (on the border between IIb and IV; Figure 6). This means that its bioavailability could be further improved by enabling formulations.

## 3. Materials and Methods

The selection of flavonoids for this investigation was performed based on the expert opinion of the authors to representatively cover the diversity of flavonoids (different subclasses of aglycones as well as their glycosides). Based on the selected list of flavonoids, a targeted search of specific properties (such as p*K_a_*, log *P*, melting point, solubility, permeability, minimal and maximal dose) was performed. The collected data were systematized in the form of tables, including the respective references (Appendix A), which were converted into graphics and discussed. Wherever it was possible, experimental values (p*K*_a_, log *P*, *T_m_*, *P*_app_, and solubility) were used; otherwise, calculated values were taken (Table A1 and Table A2). Google Scholar was used for data collection using the following combination of keywords: “name of the flavonoid and name of the property”, e.g., “apigenin” and “p*K*_a_”. Abstracts were screened for inclusion criteria. Full-text articles were used to extract experimental and calculated values.

If the maximum and/or minimum daily dose for flavonoids as dietary supplements was not reported in scientific literature, the U.S. and/or EU currently marketed dietary supplements were sources for this purpose. The maximum and/or minimum daily dose and the recommended daily intake were then used to calculate the dose-to-solubility ratio by dividing the doses by the minimum solubility.

Hansen solubility parameters for flavonoids were calculated using the Van Krevelen group contribution method. For this purpose, the HSPiP v5.1.03 software [188] was used. The molecular volume for each flavonoid was calculated using the Y-MB engine within HSPiP. The symmetry planes of molecules were calculated using the Webmo.net online tool. Flavonoid solubility parameters δD, δP, and δH were calculated by counting molecular groups in molecules (Table A3). For the construction of a Bagley plot, δH was plotted against δV, where the δV values were calculated using the following formula:

$$\delta V=\sqrt{{\delta D}^{2}+{\delta P}^{2}}$$

## 4. Conclusions

Based on the dose-to-solubility ratio and *P*_app_ values, most flavonoids are classified as poorly soluble compounds with relatively high and low permeability (Class IIa and IIb followed by Class IV respectively). Based on their p*K*_a_ and the fact that they are weakly acidic, the flavonoids reviewed should be unionized in the stomach under fasted conditions, while in fed-state (pH up to 4), only a few of them can be partially ionized. The ionization of flavonoids is expected to occur at intestinal conditions starting from the duodenum (pH approx. 5.5), following the jejunum and ileum, where the permeation should happen. In accordance with the Bagley plot, the absorption window is in the upper part of the small intestine, and a limited (up to 3 h) absorption time of all flavonoids can be expected.

Considering the mentioned absorption window and limited absorption time, formulations with Class I and III flavonoids are expected to provide immediate or fast release. In general, a lot of dietary supplements with powdered flavonoids and simple formulations (except Class I and III flavonoids) can be justifiably criticized. Albeit, based on the p*K*_a_ and log *P* of some of them, administration before/after/with a meal or with a fat meal can be used to increase their bioavailability.

Nevertheless, to decrease inter- and intra-variability, as well as food and food content effects on bioavailability to achieve more reproducible health effects, enabling formulations should be considered for Class IIa/IIb and IV flavonoids. For the Class IV flavonoids, the same approach can be used as for Class IIb; however, cellular efflux issues and first-pass metabolism should also be brought to attention.

The current review of selected flavonoids allowed us to describe their diversity, compare their properties, and assess the potential effect of these properties on biopharmaceutical consequences as well as the applicability of possible formulation strategies. A list of gaps in the flavonoid’s investigation was actualized (p*K*_a_, *P*_app_, log *P*, solubility, and recommended maximum daily dose), and an enormous potential to improve the health benefits of flavonoids through improved or enabled formulations was concluded.

## Data Availability

Not applicable.

## Appendix A

**Table A1:** Experimental and predicted/calculated values of p*K_a_*, log *P*, and melting point used for comparison of flavonoids.

Flavonoid Subgroup	Flavonoid	p*K_a_*			log *P*			Melting Point, °C (lit.)
		p*K_a1_* (exp.)	p*K_a2_* (exp.)	Chemaxon (pred.)	log *P* (exp.)	Chemaxon (pred.)	ALOGPS (pred.)	
Aglycones	Daidzein	7.01 [38]	-	6.48	2.51 [157]	2.73	3.3	323 (dec.) [161]
		7.51 [39]			2.73 [38]			
	Genistein	6.51 [38]		6.55	3.04 [157]	3.08	3.04	301.5 (dec.) [162,163]
		7.25 [39]						297–298 (slight decomposition) [161]
	Quercetin	5.81 [40]	7.12 [40]	6.38	1.82 [157]	2.16	1.81	313–314 (dec.) [162]
		6.74 [41]			1.47 [43]			316.5 [163]
		7.03 [41]			1.59 [42]			
		7.35 [33]						
		7.52 [42]						
		7.76 [43]						
		8.45 [35]						
	Myricetin	6.63 [44]	-	6.37	2.48 [44]	1.85	1.66	357–360 [164]
					2.94 [189]			
					2.76 [190]			
	Fisetin	7.42 [45]	-	6.32	1.78 [165]	1.81	2.03	330 [164]
		7.53 [33]						
		7.57 [33]						
		7.36 [33]						
	Kaempherol	6.86 [40]	8.56 [40]	6.38	1.55 [43]	2.46	1.99	276–278 [164]
		8.20 [41]			3.11 [157]			
		7.89 [33,43]						
		7.49 [46]						
		7.05 [47]						
	Taxifolin	6.68 [48]	8.89 [48]	7.74	0.79 [48]	1.82	1.07	240–242 [162]
					1.64 [166,167]			
	Baicalein	5.30 [49]	-	6.76	1.79 [20]	2.71	3.19	256–271 [164]
	Chrysin	6.44 [42]	-	6.58	2.29 [42]	3.01	3.44	285.5 [168]
		6.64 [50]						
		7.41 [33]						
		7.90 [33]						
		8.37 [35,51]						
		8.00 [33]						
	Luteolin	5.99 [52]	8.40 [52]	6.57	3.22 [157]	2.40	2.73	328–330 [164]
					1.75 [169]			
	Apigenin	8.54 [41]	9.93 [41]	6.57	1.50 [43]	2.71	3.07	347.5–352 [164]
		7.86 [43]			2.92 [157]			
		7.46 [33]						
	Diosmetin	N/A	N/A	6.58	3.10 [170]	2.55	3.06	228–230 [162]
	Hesperetin	7.06 [33]	9.92 [33]	7.86	2.60 [170]	2.68	2.52	226–228 [164]
		6.67 [43]						
	Naringenin	7.08 [33,53]	9.81 [33]	7.86	2.52 [170]	2.84	2.47	251 [171]
		6.7 [33]			2.60 [157]			
		6.80 [54]						
		7.50 [35]						
	Isoliquiritigenin	N/A	N/A	7.11	N/A	3.3	3.04	200–204 [162]
	Phloretin	7.0 [55]	-	7.96	N/A	3.9	2.23	263.5 [164]
		7.58 [53]						
	Silibinin	5.68 [30]	-	7.75	1.6 [30]	2.63	2.35	164–174 [172]
		6.63 [56]			0.73 [173]			
	Catechin	8.6 [57]	9.41 [57]	9	0.38 [43]	1.8	1.02	175 [162]
		8.64 [43]						
	Epigallocatechin	8.41 [43]	-	8.73	N/A	1.49	0.71	219–221 [171]
	Epigallocatechin gallate	7.75 [43]		7.99	1.0 [191]	3.08	2.38	140–142 [171]
	Cyanidin	5.48 [58]	6.70 [58] *	6.47	N/A	−0.8	2.41	>300 [161]
	Delphinidin	5.30 [58]	6.15 [58] *	5.98	N/A	2.77	2.07	250 (as chloride) [162]
								>350 [171]
	Malvidin	6.02 [58]	7.56 [58] *	5.30	N/A	2.83	1.98	>300 [162]
Glycosides	Daidzin	9.55 [59]	-	8.96	0.32 [157]	0.46	0.71	233–235 [162]
	Genistin	9.20 [59]	-	8.36	0.97 [157]	0.81	0.68	254–256 [162]
	Rutin	7.21 [40]	7.52 [40]	6.37	−0.64 [157]	−0.87	0.15	125 [163]
		6.33 [42]			−0.47 [42]			214–215 (dec.) [174]
	Myricitrin	N/A	-	6.43	N/A	0.60	1.18	194–197 [162,172]
	Astragalin	7.17 [40]	7.99 [40]	6.37	N/A	0.16	0.52	178 [162]
	Baicalin	4.21 [18]	8.56 [18]	2.62	1.27 [20]	0.76	1.27	202–205 (dec.) [164]
		7.6 [20]						
	Cynaroside	N/A	-	7.30	N/A	0.14	0.58	256–258 [164,171]
	Apigetrin	N/A	-	7.30	N/A	0.44	0.68	230–237 [172]
	Vitexin	6.76 [60]	8.11 [60]	6.17	N/A	0.051	0.36	256–257 [175]
	Diosmin	9.39–10.12 [61]	-	7.31	0.14 [170,176]	−0.44	0.08	277–278 [161]
	Hesperidin	10.0 [62]	-	8.61	−0.87 [177]	−0.31	−0.27	258–262 [162]
		7.75 [50]						250–255 (dec.) [164]
	Naringin	10.2 [54]	-	8.58	N/A	−0.16	−0.24	83 [171]
	Phlorizin	7.42 [53]	-	7.87	0.94 [179]	0.98	0.44	110 [162]
		7.32 [63]						113–114 [164]
	Cyanidin-3-glucoside	N/A	5.88 [58]	6.39	−1.23 [180]	0.44	0.68	>350 [178]
	Delphinidin-3-glucoside	N/A	5.35 [58]	6.37	N/A	0.93	0.10	N/A
	Malvidin-3-glucoside	1.76 [64]	5.36 [64]	6.38	−1.31 [181]	0.17	1.3	N/A

N/A—not available; exp.—experimentally determined values; pred.—predicted values, determined values by calculation; lit.—value obtained in literature; dec.—decomposition, *—predicted values.

**Table A2:** Experimental and predicted/calculated values of solubility, single dietary abundant doses, and permeability used for the comparison of flavonoids.

Flavonoid Subgroup	Flavonoid	Permeability (*P*_app_), 1 × 10^−6^ cm per s	Solubility, mg/mL		Single Dietary Dose, mg	
		Caco-2 Cells Apical to Basolateral (A to B) (exp.)	exp.	ALOGPS (pred.)	min. Value	max. Value
Aglycones	Daidzein	67.97 [38]	0.008215 [72]	0.0849	19.01 [88]	34 [89]
		21 [77]				
		36.8 [78]				
	Genistein	33.90 [79]	0.015 [90]	0.123	21.02 [88]	250 [91]
		22.50 [77]				
		3.7 [78]				
		30.9 [80]				
		52.86 [38]				
	Quercetin	3.91 [77]	0.0003 [72]	0.261	85 [92]	1600 [93]
		3.80 [42]				
		9.89 [78]				
		2.55 [79]				
		0 [81]				
	Myricetin	1.70 [77]	0.00276 (pH 3) [44]	0.301	N/A	100 [94]
		0.29 [79]				
	Fisetin	N/A	0.01045 [95]	0.151	150 [96]	1000 [97]
	Kaempherol	10.20 [77]	0.113 [98]	0.178	N/A	100 [99]
		6.68 [79]				
	Taxifolin	0.31 [77]	N/A	1.16	60 [100]	160 [101]
		6.32 [79]				
	Baicalein	7.29 [77]	0.00229 (pH 1) [102]	0.153	50 [103]	200 [104]
		1.95 [79]				
		3.51 [82]				
	Chrysin	16.00 [77]	0.003038 [105]	0.105	500 [106]	1500 [106]
		2.60 [42]				
		6.78 [79]				
	Luteolin	8.87 [77]	0.0025 [107]	0.138	N/A	100 [108]
		10.10 [79]				
		0 [81]				
	Apigenin	16.9 [80]	0.00163 [109]	0.118	50 [110]	500 [111]
		17.12 [79]				
	Diosmetin	76.2 [54]	N/A	0.0754	N/A	N/A
	Hesperetin	18.30 [77]	N/A	0.138	N/A	N/A
		47.1 [54]				
		23.50 [79]				
	Naringenin	37.8 [54]	0.00438 [112]	0.214	N/A	100 [113]
		12.60 [77]				
		32.13 [79]				
	Isoliquiritigenin	14.60 [77]	0.035 [114]	0.0551	N/A	25 [115]
		13.76 [79]				
		8.01 [82]				
	Phloretin	1.06 (10.9 µM); 0.13 (21.9 µM); 0.34 (43.8 µM) [83]	N/A	0.132	N/A	10 [116]
	Silibinin	3.2 [84]	0.055 [30]	0.0926	53 [117]	180 [118]
	Catechin	1.68 [85]	0.45 [119]	0.645	35 (as epicatechin) [120]	500 (as epicatechin) [121]
		0.14 [86]				
		0 [81]				
	Epigallocatechin	0.15 [86]	N/A	0.871	N/A	30 [120]
	Epigallocatechin gallate	0.88 [85]	52.37 (pH 5) [122]	0.0728	117 [123]	810 [124]
		0.083 [86]				
		1.35 [87]				
	Cyanidin	N/A	N/A	0.071	N/A	N/A
	Delphinidin	N/A	N/A	0.071	N/A	N/A
	Malvidin	N/A	N/A	0.023	N/A	N/A
Glycosides	Daidzin	0.0574 (detected as daidzein) [78]	0.040 [126]	0.661	3 [127]	19.25 [128]
		0.44 [77]				
	Genistin	0.0910 (detected as genistein) [78]	0.020 [126]	1.01	N/A	25 [128]
	Rutin	0 [77] 1.24 [42] 10.32 [86] 4.04 [79]	0.147 [42]	3.54	10 [129]	500 [130]
	Myricitrin	0 [77]	N/A	2.43	N/A	N/A
	Astragalin	N/A	N/A	1.58	N/A	N/A
	Baicalin	0.17 [77] 0.12 [131] 0.241 [82]	0.052 [131]	1.72	125 [103]	500 [132]
	Cynaroside	0.43 [77]	N/A	1.08	N/A	N/A
	Apigetrin	N/A	N/A	0.97	N/A	N/A
	Vitexin	N/A	0.00762 [133]	1.73	8.75 [134]	24.5 [135]
	Diosmin	Not detected [54]	0.000019 [136]	1.54	450 [137]	1000 [138]
	Hesperidin	Not detected [54] 1.11 (in rabbits cornea) [62]	0.00493 [62]	2.69	36 [139]	1000 [140]
	Naringin	Not detected [54] 3.73 [79] 0 [81]	0.087 [141]	4.06	150 [142]	500 [143]
	Phlorizin	N/A	1.93 [144]	1.21	N/A	50 [145]
	Cyanidin-3-glucoside	~0.01 (determined value from the graph) [78] 0.81 [146]	N/A	0.60	2.2 [147]	600 [147]
	Delphinidin-3-glucoside	N/A	N/A	0.85	N/A	N/A
	Malvidin-3-glucoside	N/A	N/A	0.37	N/A	N/A

N/A—not available; exp.—experimentally determined values; pred.—predicted values, determined values by calculation.

**Table A3:** Calculated flavonoid solubility parameters by group-contribution method.

Flavonoid Subgroup	Flavonoid	Groups’ Contribution													Molecular Volume, cm^3^·mol^−1^	$\delta H,\;\sqrt{\frac{J}{cm^{3}}}$	$\delta D,\;\sqrt{\frac{J}{cm^{3}}}$	$\delta P,\;\sqrt{\frac{J}{cm^{3}}}$	$\delta V,\;\sqrt{\frac{J}{cm^{3}}}$
		CH_3_	-CH_2_-	-CH<	=CH-	=C<	-C6 aromatic	-C6-aromatic *o,m,p*	-OH	-O-	-CO-	-COOH	-COO-	Ring					
Aglycones	Daidzein	-	-	-	1	1	1	1	2	1	1	-	-	1	201.40	15.00	21.60	5.40	22.26
	Genistein	-	-	-	1	1	1	1	3	1	1	-	-	1	211.50	17.60	21.60	5.80	22.37
	Quercetin	-	-	-	-	2	1	1	5	1	1	-	-	1	219.80	21.90	22.10	6.50	21.32
	Myricetin	-	-	-	-	2	1	1	6	1	1	-	-	1	234.90	23.10	21.50	6.40	20.90
	Fisetin	-	-	-	-	2	1	1	4	1	1	-	-	1	204.30	20.40	22.70	6.50	21.89
	Kaempherol	-	-	-	-	2	1	1	4	1	1	-	-	1	211.30	20.10	22.00	6.30	21.16
	Taxifolin	-	-	2	-	-	1	1	5	1	1	-	-	1	232.20	21.30	21.80	5.90	20.85
	Baicalein	-	-	-	1	1	1	1	3	1	1	-	-	1	208.90	17.60	21.70	5.60	20.58
	Chrysin	-	-	-	1	1	1	1	2	1	1	-	-	1	200.50	15.00	21.40	6.00	20.59
	Luteolin	-	-	-	1	1	1	1	4	1	1	-	-	1	222.40	19.50	21.60	5.80	20.63
	Apigenin	-	-	-	1	1	1	1	3	1	1	-	-	1	211.50	17.50	20.50	5.70	21.28
	Diosmetin	1	-	-	1	1	1	1	3	2	1	-	-	1	229.60	17.20	22.70	5.40	20.62
	Hesperetin	1	1	1	-	-	1	1	3	2	1	-	-	1	239.70	16.80	20.50	5.20	20.19
	Naringenin	2	2	11	-	-	1	1	8	6	1	-	-	3	220.00	17.20	22.10	6.50	21.32
	Isoliquiritigenin	-	-	-	2	-	-	2	3	-	1	-	-	-	197.50	17.70	21.50	5.90	20.37
	Phloretin	-	2	-	-	-	-	2	4	-	1	-	-	-	204.30	20.00	22.50	6.20	21.51
	Silibinin	1	1	4	-	-	2	1	5	4	1	-	-	2	367.50	17.60	19.80	4.30	20.26
	Catechin	-	1	2	-	-	1	1	5	1	-	-	-	1	232.10	21.10	20.60	5.20	19.99
	Epigallocatechin	-	1	2	-	-	1	1	6	1	-	-	-	1	254.80	22.00	28.80	6.10	19.09
	Epigallocatechin gallate	-	1	2	-	-	1	2	8	1	-	-	1	1	324.90	22.90	22.30	4.80	21.35
	Cyanidin	-	-	-	-	-	-	-	-	-	-	-	-	-	N/A	N/A	N/A	N/A	N/A
	Delphinidin	-	-	-	-	-	-	-	-	-	-	-	-	-	N/A	N/A	N/A	N/A	N/A
	Malvidin	-	-	-	-	-	-	-	-	-	-	-	-	-	N/A	N/A	N/A	N/A	N/A
Glycosides	Daidzin	-	1	5	1	1	1	1	5	3	1	-	-	2	315.80	18.70	19.10	4.90	18.53
	Genistin	-	1	5	1	1	1	1	6	3	1	-	-	2	350.00	19.30	17.90	4.60	17.42
	Rutin	-	1	10	-	-	1	1	10	5	1	-	-	3	520.40	20.40	17.00	3.80	15.18
	Myricitrin	1	-	5	-	2	1	1	8	3	1	-	-	2	396.50	20.80	17.00	4.60	16.50
	Astragalin	-	-	-	-	2	1	1	7	3	1	-	-	2	360.30	20.50	17.60	4.70	16.10
	Baicalin	-	-	5	1	1	1	1	5	3	1	1	-	2	317.00	19.50	19.40	5.00	19.36
	Cynaroside	-	1	5	1	1	1	1	7	3	1	-	-	2	360.40	20.50	17.90	4.70	17.54
	Apigetrin	-	1	5	1	1	1	1	6	3	1	-	-	2	350.00	19.30	17.90	4.60	17.42
	Vitexin	-	1	5	1	1	1	1	7	3	1	-	-	2	328.10	21.50	19.40	5.00	19.19
	Diosmin	2	1	10	1	1	1	1	8	6	1	-	-	3	518.50	18.50	15.30	3.60	15.72
	Hesperidin	1	3	10	-	-	1	1	9	4	1	-	-	2	529.90	18.40	15.60	3.60	15.72
	Naringin	-	2		-	-	-	2	4	-	1	-	-	-	508.60	19.50	15.90	3.70	15.55
	Phlorizin	-	3	5	-	-	-	2	7	1	1	-	-	-	360.00	20.10	17.40	4.60	16.21
	Cyanidin-3-glucoside	-	-	-	-	-	-	-	-	-	-	-	-	-	N/A	N/A	N/A	N/A	N/A
	Delphinidin-3-glucoside	-	-	-	-	-	-	-	-	-	-	-	-	-	N/A	N/A	N/A	N/A	N/A
	Malvidin-3-glucoside	-	-	-	-	-	-	-	-	-	-	-	-	-	N/A	N/A	N/A	N/A	N/A

N/A—not applicable because the compound has an ionic nature.
